# Radiochromic film based dosimetry of image‐guidance procedures on different radiotherapy modalities

**DOI:** 10.1120/jacmp.v15i6.5006

**Published:** 2014-11-08

**Authors:** Ahmad Nobah, Saad Aldelaijan, Slobodan Devic, Nada Tomic, Jan Seuntjens, Mohammad Al‐Shabanah, Belal Moftah

**Affiliations:** ^1^ Biomedical Physics Department King Faisal Specialist Hospital & Research Centre Riyadh Saudi Arabia; ^2^ Department of Radiation Oncology Jewish General Hospital Montréal Québec Canada; ^3^ Medical Physics Unit McGill University Montréal Québec Canada

**Keywords:** radiochromic film dosimetry, IGRT, imaging dose

## Abstract

In this work we compare doses from imaging procedures performed on today's state‐of‐the‐art integrated imaging systems using a reference radiochromic film dosimetry system. Skin dose and dose profile measurements from different imaging systems were performed using radiochromic films at different anatomical sites on a humanoid RANDO phantom. EBT3 film was used to measure imaging doses from a TomoTherapy MVCT system, while XRQA2 film was used for dose measurements from kilovoltage imaging systems (CBCT on 21eX and TrueBeam Varian linear accelerators and CyberKnife stereoscopic orthogonal imagers). Maximum measured imaging doses in cGy at head, thorax, and pelvis regions were respectively 0.50, 1.01, and 4.91 for CBCT on 21eX, 0.38, 0.84, and 3.15 for CBCT on TrueBeam, 4.33, 3.86, and 6.50 for CyberKnife imagers, and 3.84, 1.90, and 2.09 for TomoTherapy MVCT. In addition, we have shown how an improved calibration system of XRQA2 film can achieve dose uncertainty level of better than 2% for doses above 0.25 cGy. In addition to simulation‐based studies in literature, this study provides the radiation oncology team with data necessary to aid in their decision about imaging frequency for image‐guided radiation therapy protocols.

PACS number: 87.53.Bn, 87.55.Qr, 87.56.Fc

## I. INTRODUCTION

Image‐guided radiotherapy (IGRT) has become standard of care in advanced highly conformal radiotherapy techniques, mainly because of advantages it offers in improving target localization by minimizing errors in patient positioning prior to treatment delivery.^1^, ^2^, ^3^ However, the increased imaging sessions associated with IGRT posed questions about the amount of concomitant dose burden.^4^, ^5^ The American Association of Physicists in Medicine (AAPM) Task Group 75 has differentiated between the management of imaging dose from IGRT and doses received from diagnostic radiology exams because IGRT doses could be optimized within treatment regimen.^6^, ^7^ The importance of imaging dose management in IGRT stems not only from concepts such as ALARA (as low as reasonably achievable), but it also constitutes a risk of stochastic effects outside the primary treatment area which is in parallel to risks arising from linear accelerator head leakage and scatter.^(4)^ Furthermore, realization of this risk becomes especially important in the course of whole treatment when one considers possibility of approaching deterministic effects borderlines from increased dose to organs at risk.^4^, ^8^

Patient dose from imaging in IGRT has been assessed for various imaging systems using different dosimeters. This includes point dosimeters such as ionization chambers,^9^, ^10^, ^11^ thermoluminscent detectors (TLD),^4^, ^7^, ^12^ MOSFETs,^(9)^ optically stimulated luminescence dosimeters (OSLD),^(13)^ radiochromic films used as either point dosimeters^13^, ^14^ or as 2D dosimeters,^15^, ^16^ as well as Monte Carlo (MC) simulations.^5^, ^8^, ^17^ Most of the aforementioned studies reported doses measured to the head, chest, and pelvic regions in phantoms,^4^, ^5^, ^8^, ^9^, ^10^, ^13^, ^15^, ^17^, ^18^, ^19^, ^20^ and only few in selected patients.^9^, ^11^, ^18^

Radiochromic film has been used extensively in dosimetry of kilovoltage (kV) and megavoltage (MV) beams. For kV beams, XRQA and XRQA2 GAFCHROMIC film models were used by several groups to measure doses delivered during kV cone‐beam computed tomography (CBCT)^13^, ^15^, ^19^ and diagnostic CT,^12^, ^21^, ^22^ both sharing the same dosimetry method. Tomic et al.^15^, ^16^ used XRQA GAFCHROMIC film model calibrated in terms of air kerma in air to measure on‐board imaging (OBI) CBCT imaging dose profiles and surface doses for head and neck, thorax, and pelvis of a humanoid RANDO phantom. They have shown that, for typical kV CBCT beam qualities, simple mass‐energy absorption coefficient ratios water‐to‐air can approximate the dose conversion from measured by film air kerma in air at the surface to a dose to water at the phantom surface, following the AAPM TG‐61 protocol.^(23)^ Giaddui et al.^(21)^ investigated different characteristics of XRQA2 GAFCHROMIC film model for dosimetry of kV photon beams. This included scanning mode, film size, region of interest size, scan uniformity, scan resolution, scan orientation, energy dependence, and reflectance postirradiation evolution with time. For MV beams, EBT, EBT2, and EBT3 GAFCHROMIC film models were used extensively to measure therapeutic doses over wide dose range and the same dosimetry protocols apply for imaging. The dosimetric properties of these films were tested widely in the literature in terms of absorption spectra,^(24)^ postirradiation waiting time,^24^, ^25^ scanning mode and exposure to light,^25^, ^26^ high temperature behavior,^(25)^ performance in water,^(27)^ depth dose measurements,^(28)^ and energy dependence.^29^, ^30^

To the best of our knowledge, all studies found in the literature reported either point doses using a single imaging tool^8^, ^9^, ^10^, ^14^, ^17^ (or different imaging tools^4^, ^5^, ^11^, ^13^), or 2D profiles of a single imaging tool.^12^, ^15^, ^16^ The only study that compared different patient profiles from multiple imaging tools using the same dosimetry method was the recent MC simulations by Ding and Munro^(5)^ where they compared imaging doses from kV CBCT, orthogonal kV radiographs, and MV portal images. In this study, we compare measured imaging doses delivered by different IGRT imaging systems in terms of 2D dose profiles and surface doses within head, chest, and pelvic region using the same reference radiochromic film dosimetry system.^16^, ^18^ It was already reported^(31)^ that choice of dosimeter and consequently corresponding effective point of measurement could have a significant impact on measured surface doses. In this regards, our work provides a more reliable comparison method by using the same dosimeter.

## II. MATERIALS AND METHODS

### A. IGRT systems

In this study, we compare four different IGRT imaging systems used in contemporary radiotherapy: cone‐beam computed tomography (CBCT) system mounted on Varian 21eX and TrueBeam clinical linear accelerators (Varian, Palo Alto, CA) as a part of On‐Board Imaging (OBI) system; TomoTherapy Hi·ART (Accuray Inc., Sunnyvale, CA) fan beam megavoltage computed tomography (MVCT); and 2D kV stereoscopic orthogonal imagers on CyberKnife treatment unit (Accuray Inc.).

#### A.1 Varian OBI cone‐beam CT system

**Figure 1:**
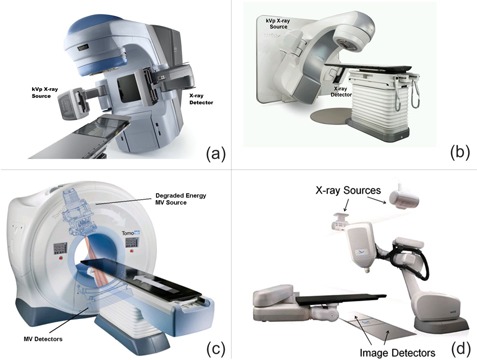
Four IGRT systems investigated in this work: a) OBI system on 21eX Varian linear accelerator; b) OBI system on Varian TrueBeam linear accelerator; c) megavoltage CT (MVCT) on TomoTherapy system; d) two orthogonal radiographs imaging on CyberKnife system.

Retractable kV source (KVS) operates in energy range of 100–125 kVp and flat‐panel amorphous silicon detector is mounted on the 21eX (Fig.1(a)) and TrueBeam (Fig.1(b)) linacs. The system is mounted orthogonally to the treatment beam axis and can acquire 2D radiography, fluoroscopy, and 3D CBCT images. On the earlier OBI system (attached to 21eX, Fig.1(a)), two aluminum filters (full and half bowtie) can be inserted in front of the KVS to harden the beam, make the photon fluence uniform across the detector, and reduce the scatter and patient skin dose.^5^, ^15^, ^32^ The latest OBI systems installed on TrueBeam linacs have bowtie filters embedded into the KVS housing, which is more convenient for operators, and they also contain Titanium which further hardens the beam without image quality detriment and further decreases skin dose.^(5)^

#### A.2 TomoTherapy MV fan‐beam CT system

Helical tomotherapy system (Fig.1(c)) utilizes MV beams to implement the tomography methodology into radiotherapy for both imaging and treatment. TomoTherapy machine has two modes of operation: 1) treatment mode when 6 MV beam is used to deliver prescribed dose distribution to patients, and 2) imaging mode where a degraded 3.5 MeV beam is used with a fan‐shaped array of detectors mounted opposite to the MV source.^(33)^ In this work (as well as in our clinic) we used the standard pitch value of 2 resulting in slice thickness of 4 mm. The default image reconstruction matrix size is 512×512, with 40 cm field of view.

#### A.3 CyberKnife 2D orthogonal IGRT system

CyberKnife (CK) is fully integrated image‐guided, frameless, robotic radiosurgery system (Fig.1(d)) composed of a compact 6 MV X‐band linear accelerator mounted on mobile robotic arm and kV X‐ray imaging system that can synchronize movement of the robot accordingly as the target location is updated during delivery process.^(34)^ Inside the CyberKnife treatment room, two image‐guidance systems are rigidly fixed — two imaging sources (A & B) are mounted to the ceiling and two imaging detectors are installed on the floor. The nominal square field at the patient level can be $17\times 17\;{\text{cm}}^{2}$ and the KVS imaging parameters range between 40–125 kVp, 25–300 mA, and 1–500 ms. The radiation treatment can be guided by different tracking modes either by using static or dynamic image acquisition, depending on the site and location of the target and its surrounding anatomical structures.^(35)^

### B. Radiochromic film dosimetry system

For dose measurements on RANDO phantom (The Phantom Laboratory, Salem, NY) receiving dose from TomoTherapy unit, we used the EBT3 model GAFCHROMIC film (Ashland, Wayne, NJ) with Lot number: A03181302. Film pieces were calibrated in Solid Water phantom using 4 MV photon beam from the 21eX linac for doses up to 100 cGy, following previously established reference radiochromic film dosimetry protocol,^(36)^ with the idea to perform multiple imaging acquisitions on RANDO phantom and scale measured doses to report results for one image acquisition.

Imaging dose measurements on remaining three systems (OBI and CK) were performed using the XRQA2 model GAFCHROMIC film, lot number: A10071002B. Response of the film dosimetry system was calibrated in terms of air kerma in air up to 10 cGy, following previously established dosimetry protocol.^15^, ^18^ Change in reflectance of measuring film pieces placed within RANDO phantom were converted into air kerma in phantom using the calibration curve and then into dose to water, following TG‐61 protocol,^(23)^ which resulted in simple multiplication by the ratio of mass‐energy absorption coefficient water to air (introducing an error in the film dosimetry system of less than 1%).^(15)^

For both film models, response of the film was determined from images of the film pieces scanned prior and 24 hrs postirradiation. All films were scanned with an image resolution of 0.2 mm/pixel in 48 bit RGB mode (16 bits per color). Change in net reflectance (netΔR) for XRQA2 or change in net optical density (netΔOD) for EBT3 film model were obtained from scanned images using an in‐house image MATLAB routine (MathWorks, Inc., Natick, MA). For a given film piece, the pixel values were sampled over the same region of interest (ROI) on two images (before and after the exposure). Film reflectance and/or optical density were calculated using pixel values (PV) from the red color channel sampled over $1\times 1\;{\text{mm}}^{2}$ ROI.

**Figure 2:**
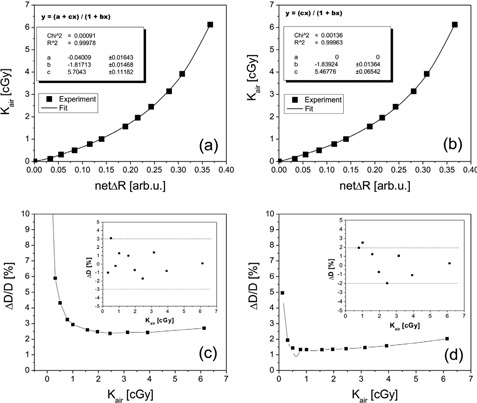
Calibration curves for “old” model (a) and “new” improved model (b) used to calibrate XRQA2 model GAFCHROMIC film based dosimetry system; (c) and (d) represent the total one sigma dose uncertainty (subplots) as a function of air kerma in air together with dose error analysis in percentage (insets) of calibration models in (a) and (b), respectively.

In our previous work,^(18)^ measured change in reflectance as a function of air kerma in air was fitted using the rational function y=(a+cx)/(1+bx), resulting in an uncertainty of 3% or lower for doses above 3 cGy (Figs.2(a) and (c)). In this work, we used slightly modified function, by forcing the parameter, a, to be zero during fitting process (Figs. 2(b) and (d)). The “old” fitting function represents mathematically more general rational function form and, while giving result for the fitting parameter a close to zero, the associated fitting uncertainty is relatively large (41%), resulting in relatively large total uncertainty at lower doses. By forcing the parameter, a, to be zero in the “new” fitting function, remaining fitting parameters (b and c) remain almost identical with a clear gain in reduced uncertainty, particularly at low‐dose values. Setting parameter, a, to zero during fitting procedure can be easily justified, as physically we expect that, for zero dose point, change in net reflectance should be zero as well. Uncertainty analysis graphs shown at the bottom of Fig. 2 indicate that the “new” calibration function improved uncertainty of measured dose from 3% for doses above 1 cGy to better than 2% for doses above 0.25 cGy.

### C. Dose measurements

Imaging dose measurements were performed for three different body regions: head, thorax, and pelvis using humanoid RANDO phantom. Summary of imaging protocols used on different IGRT systems are given in Table 1. Imaging parameters on every machine were chosen based on IGRT protocols used clinically. Surface dose measurements during imaging acquisitions were performed by taping 1“ by 2“ film pieces on surface of RANDO phantom at the slice in the center of imaging volume. Also, 1” wide film strips were placed between RANDO slabs in both vertical and lateral directions to measure dose profiles during imaging scans. In order to minimize air gaps between slices, two film strips were inserted between two neighboring slabs of the RANDO phantom. All measurements for all imaging protocols listed in Table 1 were repeated three times and the average doses were reported.

**Table 1:** Different IGRT imaging protocols on four different commercially available systems.

*Anatomical Modality*	*Site*	*Peak Acquisition Technique*	*Beam Energy*	*Total Quality*	*mAs / MUs*
Varian 21eX	Head	Standard dose, FF (200o)	100 kV	4.9 mm Al	145
Varian 21eX	Thorax	Low Dose Thorax, HF (360o)	110 kV	5.2 mm Al	262
Varian 21eX	Pelvis	Pelvis, HF (360o)	125 kV	5.8 mm Al	1049
TrueBeam	Head	Standard dose, FF (200o)	100 kV	6.9 mm Al	146
TrueBeam	Thorax	Thorax Slow, HF (360o)	125 kV	7.7 mm Al	252
TrueBeam	Pelvis	Pelvis, HF (360o)	125 kV	7.7 mm Al	1056
CyberKnife	Head	6D Skull / 100 image pairs	100 kV	5.4 mm Al	1250
CyberKnife	Thorax	C‐spine / 100 image pairs	110 kV	5.7 mm Al	1500
CyberKnife	Pelvis	L‐Spine / 100 image pairs	125 kV	6.4 mm Al	4000
TomoTherapy	Head	Pitch 2 (TG 148)	3.5 MeV	3.5 MV	6.4 MU
TomoTherapy	Thorax	Pitch 2 (TG 148)	3.5 MeV	3.5 MV	6.4 MU
TomoTherapy	Pelvis	Pitch 2 (TG 148)	3.5 MeV	3.5 MV	6.4 MU

Since we expected relatively small measured doses, each imaging dose measurement was performed by carrying multiple scans and report results per single scan: for the OBI system we performed five scans; for the TomoTherapy system we performed five identical MVCT scans. For the CyberKnife system, we took 100 image pairs (200 images) and report results per 100 image pairs (corresponding to common number of image pairs in CyberKnife treatments).

## III. RESULTS & DISCUSSION

Table 2 lists obtained surface doses together with their standard errors (expressed as one sigma uncertainties) obtained from four different IGRT systems within different anatomical regions of RANDO phantom. Within head, the OBI system exhibits its highest dose at the posterior and lowest at the anterior aspect of the skull. This is due to the acquisition geometry used (see Table 1) whereby X‐ray tube rotates around posterior aspect of the patient. In addition, OBI system installed on TrueBeam shows further decrease in surface dose due to addition of Titanium into bowtie filters, while mAs setting for both OBI versions remained the same. For CyberKnife system, we observed the opposite trend whereby the anterior dose is the highest, which is explained by the fact that sources are mounted above the patient. Finally, surface doses measured from TomoTherapy unit appear to be more or less uniform due to full helical nature of the MVCT acquisition. Slightly higher dose observed on the posterior aspect of the phantom can be explained by creation of charged particles within treatment couch that land directly onto posterior surface of the phantom.

**Table 2:** Surface doses measured (in cGy) using RANDO phantom for different IGRT protocols on four different commercial systems.

*Modality*		*OBI – 21eX*	*OBI – TrueBeam*	*CyberKnife*	*TomoTherapy*
*Surface Dose [cGy]*					
Head	Ant	0.05±0.01	0.09±0.01	4.33±0.07	2.07±0.03
	Post	0.50±0.01	0.27±0.01	0.50±0.01	2.92±0.05
	Left	0.38±0.01	0.32±0.01	2.27±0.04	2.22±0.04
	Right	0.44±0.01	0.31±0.01	2.09±0.04	2.31±0.04
Thorax	Ant	0.75±0.01	0.65±0.01	3.86±0.06	1.05±0.02
	Post	0.64±0.01	0.48±0.01	0.45±0.01	1.70±0.03
	Left	0.58±0.01	0.47±0.01	1.75±0.03	0.97±0.02
	Right	0.65±0.01	0.51±0.01	1.74±0.03	0.89±0.02
Pelvis	Ant	3.72±0.06	3.10±0.05	6.50±0.10	0.98±0.02
	Post	3.48±0.05	2.02±0.03	0.30±0.01	1.49±0.03
	Left	2.74±0.04	1.89±0.03	3.30±0.05	0.79±0.02
	Right	2.58±0.04	2.02±0.03	3.25±0.05	0.71±0.02

For the imaging protocols within thorax region, the OBI systems use slightly harder beams (to accommodate larger patient volume) and larger mAs setting. In addition, acquisition geometry uses a full rotation and the measured doses are slightly higher than in the case of head region. Surface doses measured on CyberKnife system are slightly lower than in the case of head protocol despite slight increase in mAs notably due to the increased size of the imaging volume. Contrary to kVp imaging systems, TomoTherapy system exhibits decrease in measured surface dose due to the fact that acquisition parameters (beam quality, pitch, output (equivalent to number of MUs)) remained the same and the size of the imaging volume increased.

Finally, surface doses measured for OBI systems in pelvis region further increases as compared to head and thorax region as a result of almost a fourfold increase in the mAs setting. For CyberKnife, surface dose measurements show further increase with the same trend having the highest anterior dose while, for TomoTherapy, unit surface doses remained approximately unchanged since the output (equivalent to number of MUs) and beam quality did not change.

**Figure 3:**
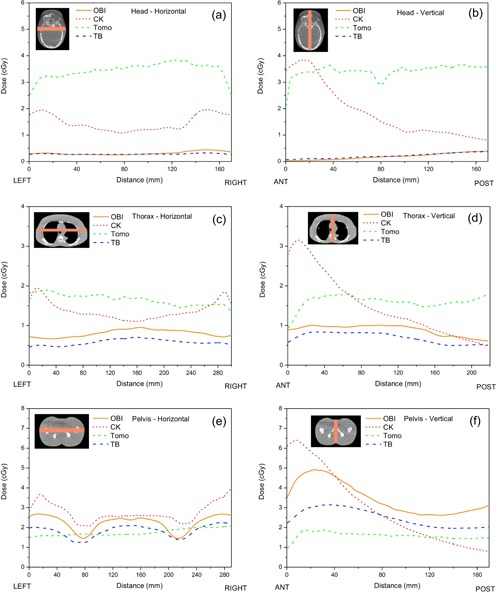
Horizontal and vertical dose profiles obtained through RANDO phantom in the head and neck region ((a) and (b)), thorax ((c) and (d)), and pelvis region ((e) and (f)) on four different IGRT systems: OBI CBCT on Varian 21eX linear accelerator (orange solid lines), CyberKnife imagers (red dotted lines), Tomotherapy MVCT (green dot‐dashed lines), and OBI CBCT on Varian TrueBeam linear accelerator (blue dashed lines).

The obtained dose profiles from different body regions and IGRT systems are presented in Fig. 3. Lateral profiles (Figs. 3(a), (c), and (e)) exhibit a slight asymmetry with respect to the central axis of the imaging systems for OBI and TomoTherapy units, while they appear to be symmetric for the CyberKnife system. This is a consequence of the fact that, for the former two systems, patients are usually positioned for the actual treatment in such a way that the target is placed in the isocenter of the machine, whereas the midplane of the patient is offset by the amount of the iso‐shift for a given plan. To simulate actual clinically relevant situations, in our measurements we aligned RANDO phantom using fiducial marks corresponding to actual treatment positions which, in turn, results in slightly asymmetric lateral profiles.

On the other hand, vertical profiles (Figs. 3(b), (d), and (f)) exhibit quite large asymmetry for the CyberKnife which is a consequence of the fact that X‐ray sources are mounted above the patient. Significant asymmetry in case of OBI system is observed for the head section only, whereby the X‐ray tube rotates below the patient. Rationale for such setup is to minimize dose to the more radiosensitive structures at the anterior side. Relatively small asymmetry for other imaging modalities exhibiting higher doses at the anterior aspect within thorax and pelvis regions can be explained by the presence of the treatment couch that may attenuate slightly the beam used for imaging.

Results of surface dose measurements and dose profiles suggest relatively large variation in both magnitude and dose distributions within RANDO phantom and, hence, patient body. These variations are governed mainly by different factors such as beam quality, mAs, source‐to‐surface distance (SSD), field size, detector size, system version,^16^, ^18^ anatomical site, and geometry of acquisition. Therefore, imaging techniques or protocols, together with different setups, arguably result in different reported doses in the literature and comparison of these results must be restricted to studies with similar acquisition parameters. For this study (and literature between brackets, summarized in Table 3), imaging doses in cGy at head, thorax, and pelvis ranged anywhere between 0.03–0.50 (0.05–0.47), 0.58–1.01 (0.42–0.64), 1.45–4.91 (1.6–2.64) for OBI CBCT on 21eX;^5^, ^16^ 0.07–0.38 (0.04–0.74), 0.46–0.84 (0.2–0.98), 1.24–3.15 (1.0–3.48) for OBI CBCT on TrueBeam;^5^, ^13^, ^20^ 0.50–4.33 (2.2–7.0), 0.45–3.86 (2.5–5.0), 0.30–6.50 (2.5–9.1) for CyberKnife imagers;^6^, ^14^ and 2.07–3.84 (0.5–1.76), 0.89–1.90 (1.04–1.76), 0.71–2.09 (1.01–1.35) for TomoTherapy MVCT,^10^, ^17^ respectively. As pointed out earlier, when comparing dose measurements between published data, one has to take into account the effective point of measurement for a given dosimeter. One should also keep in mind that our reported dose values are expressed in terms of dose to water.^16^, ^19^ In order to estimate dose to an organ, measured doses can be converted by the appropriate ${(\mu_{\text{en}}/{\rho )}^{\text{tissue}}}_{\text{water}}$ factor, from the AAPM TG‐61 document, at points corresponding to different tissues along measured dose profiles.

**Table 3:** Summary of reported imaging doses in the literature compared to this study (between brackets). One must take into account the geometric differences when comparing dose measurements between published data.

*Modality*	*Anatomical Site*	*Studies*	*Dose Range From Literature in cGy (this study)*
OBI CBCT on Varian 21eX	Head	Tomic et al. 2010	0.03–0.50 (0.05–0.47)
OBI CBCT on Varian 21eX	Thorax	Ding et al. 2013	0.58–1.01 (0.42–0.64)
OBI CBCT on Varian 21eX	Pelvis		1.45–4.91 (1.6–2.64)
OBI CBCT on Varian TrueBeam	Head	Chang et al. 2012	0.07–0.38 (0.04–0.74)
OBI CBCT on Varian TrueBeam	Thorax	Giaddui et al. 2013	0.46–0.84 (0.2–0.98)
OBI CBCT on Varian TrueBeam	Pelvis	Ding et al. 2013	1.24–3.15 (1.0–3.48)
CyberKnife imagers	Head	Murhpy et al. 2007	0.50–4.33 (2.2–7.0
CyberKnife imagers	Thorax	Antypas et al. 2008	0.45–3.86 (2.5–5.0)
CyberKnife imagers	Pelvis		0.30–6.50 (2.5–9.1)
TomoTherapy MVCT	Head	Meeks et al. 2005	2.07–3.84 (0.5–1.76)
TomoTherapy MVCT	Thorax	Shah et al. 2008	0.89–1.90 (1.04–1.76)
TomoTherapy MVCT	Pelvis		0.71–2.09 (1.01–1.35)

Table 4 summarizes number of treatment fractions and imaging frequency used for IGRT protocols in our department on various treatment systems. While our results suggest that imaging dose on CyberKnife system appears to be the highest, the number of treatment fractions on this system is at least five times smaller than on other radiotherapy machines. The AAPM TG‐75 document does not recommend simple summation of imaging and treatment doses,^(6)^ but it suggests effective doses to be added. While effective doses during CT examination could be estimated using the AAPM TG‐23 and TG‐204 documents,^37^, ^38^ accurate assessments of effective doses to patients from MV beams are not yet readily available in clinics. On the other hand, imaging dose from the TomoTherapy imaging system can be directly subtracted from prescription dose as both beam qualities do not differ significantly in terms of radiobiological effects. Hence, at this moment, only in the case of TomoTherapy unit, imaging dose could be offset by adding it to the prescription dose.^(17)^

**Table 4:** IGRT frequency for clinical protocols used in our clinic. “fr” stands for fraction and “W” stands for week number.

		*OBI*		*TomoTherapy*	*Cyber Knife*		
*Anatomical Site*	*# fr*.	*2D kV Orthogonal Radiographs*	*kV CBCT*	*MVCT*	*# fr*.	*2D kV Orthogonal Radiographs*	*# images/fr*
Head and Neck	25–35	Daily	W 1: First 3 fr, W 2, 3, 4: 1/week	Daily	1–5	Daily	60–80
Lung	30–35	Daily	W 1: First 3 fr, W 2, 3, 4: 1/week	Daily	4	Daily	80–100
Prostate	28–40	Daily	W 1: First 3 fr, W 2, 3, 4: 2/week	Daily	5	Daily	60–80

## IV. CONCLUSIONS

In this work, we compared the imaging doses delivered to three anatomical sites (head, thorax, and pelvis) of a humanoid RANDO phantom undergoing IGRT procedures from four different IGRT systems, using an improved reference radiochromic film dosimetry system. We found that, for typical clinical applications, the imaging dose can vary between different imaging systems. These variations are governed by anatomical site imaged, geometry of the acquisition protocols, and imaging techniques used. In addition to previously published Monte Carlo simulation‐based comparative study, our results provide the radiation oncology teams with information about measured imaging doses that may help in making decision about imaging frequency during the course of radiotherapy treatment.

## ACKNOWLEDGMENTS

This work was partially supported by the Natural Sciences and Engineering Research Council of Canada contract No. 386009. Slobodan Devic is Senior Research Scientist supported by the Fonds de Recherche en Santé du Québec (FRSQ).
